# Mechanisms and Implications of CDK4/6 Inhibitors for the Treatment of NSCLC

**DOI:** 10.3389/fonc.2021.676041

**Published:** 2021-07-30

**Authors:** Jinmeng Zhang, Dayu Xu, Yue Zhou, Zhengfei Zhu, Xi Yang

**Affiliations:** ^1^Department of Radiation Oncology, Fudan University Shanghai Cancer Center, Shanghai, China; ^2^Department of Oncology, Shanghai Medical College, Fudan University, Shanghai, China

**Keywords:** cyclin D-dependent kinase 4/6 inhibitor, cell cycle, NSCLC, therapy, drugs

## Abstract

Cyclin-dependent kinases (CDKs) are key regulators of cell cycle progression in malignant tumor cells and play an important role through complex molecular interactions. Dysregulation of CDK dependent pathways is often found in non-small cell lung cancer, which indicates its vulnerability and can be used in clinical benefit. CDK4/6 inhibitors can prevent tumor cells from entering the G approved 1 and S phases, which have been studied in a series of explorations and brought great clinical effect to patients and encouragement to both physicians and researchers, thereby showing potential as a new therapeutic agent. A series of preclinical and clinical studies have been carried out on CDK4/6 inhibitors in NSCLC, and have been achieved some results, which may become a new potential treatment in the future. This review focuses on the research progress on CDK4/6 inhibitors in NSCLC, particularly the mechanisms of action, drugs, clinical research progress, and future application.

## Introduction

Lung cancer is one of the frequently diagnosed cancer and is among the main causes of cancer death. Non-small cell cancer (NSCLC) accounts for approximately 85% of lung malignancies. Most of newly diagnosed patients are considered incurable because of the presence of metastases at the time of initial presentation (1). Despite a growing number of treatment methods for advanced NSCLC, the overall benefit is limited. Novel therapeutic targets for NSCLC have attracted considerable interest.

**Figure 1:**
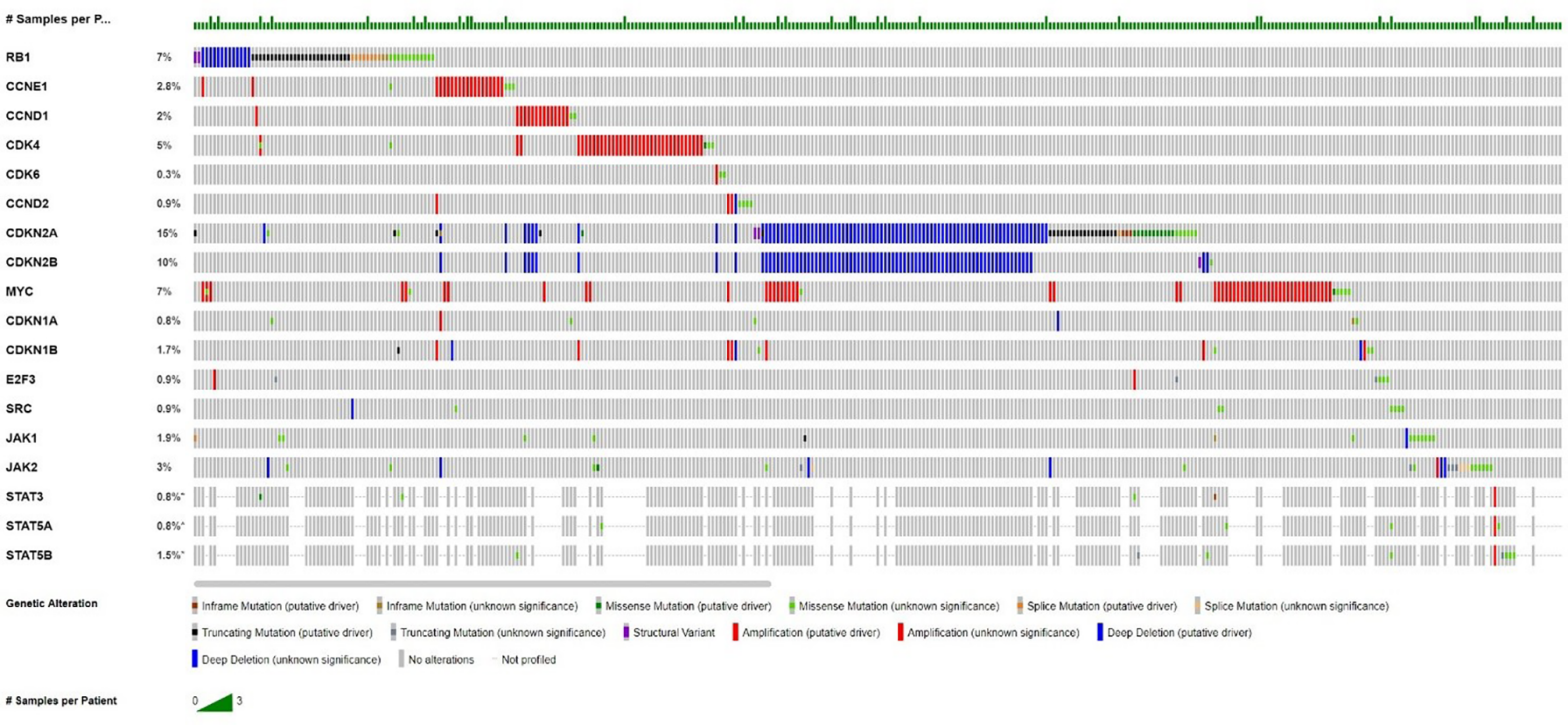
Frequencies of aberrations in the Cyclin D-CDK4/6-Rb pathway related genes in NSCLC.

In normal and malignant cells, cyclin dependent kinases (CDKs) are the key regulators, which play roles in multiple points in the cell cycle to drive cellular proliferation through most complex molecular interactions (2). The expression and activation of cell cycle mediators is deranged, especially within the CDK–cyclin–RB pathways, and is involved in malignant transformation and tumor progression in lung cancer (3). In over 90% of lung cancers, the cell cycle occurs as dysregulation, which makes the derangements of cell cycle mediators in the expression and/or activation, especially within the CDK–cyclin–RB pathways, and is integrally involved in malignant transformation and tumor progression, destroying the cell proliferation mechanism controlling the growth of advanced NSCLC (3–5). Cyclin-dependent kinases 4 and 6 (CDK4 and CDK6) form a complex with D-type cyclins, which promote the cell cycle through the G1 restriction point through phosphorylation of the Rb tumor suppressor protein (6). Inhibition of CDK4 and CDK6 can prevent cell cycle progression, prevent tumor growth and promote senescence. Through aberrant retinoblastoma protein (RB) expression and the mutations of cyclin D CDK4 (INK4) proteins (p16INK4A) (7) and K-RAS, these cyclins promote uncontrolled cellular proliferation and drive cell cycle progression in lung carcinogenesis (8). According to the analysis of UALCAN cancer database, the CDK6 gene was moderately expressed in LUAD, and the overall survival rate of patients was negatively correlated with it. **Figure 1** shows frequencies of aberrations in the Cyclin D-CDK4/6-Rb pathway related genes in NSCLC from the publicly available cBioportal webpage: Pan-Lung Cancer (TCGA, Nat Genet 2016). Therefore, CDK4/6 has been a key target for the clinical development for cancer therapy (9, 10).

**Figure 2:**
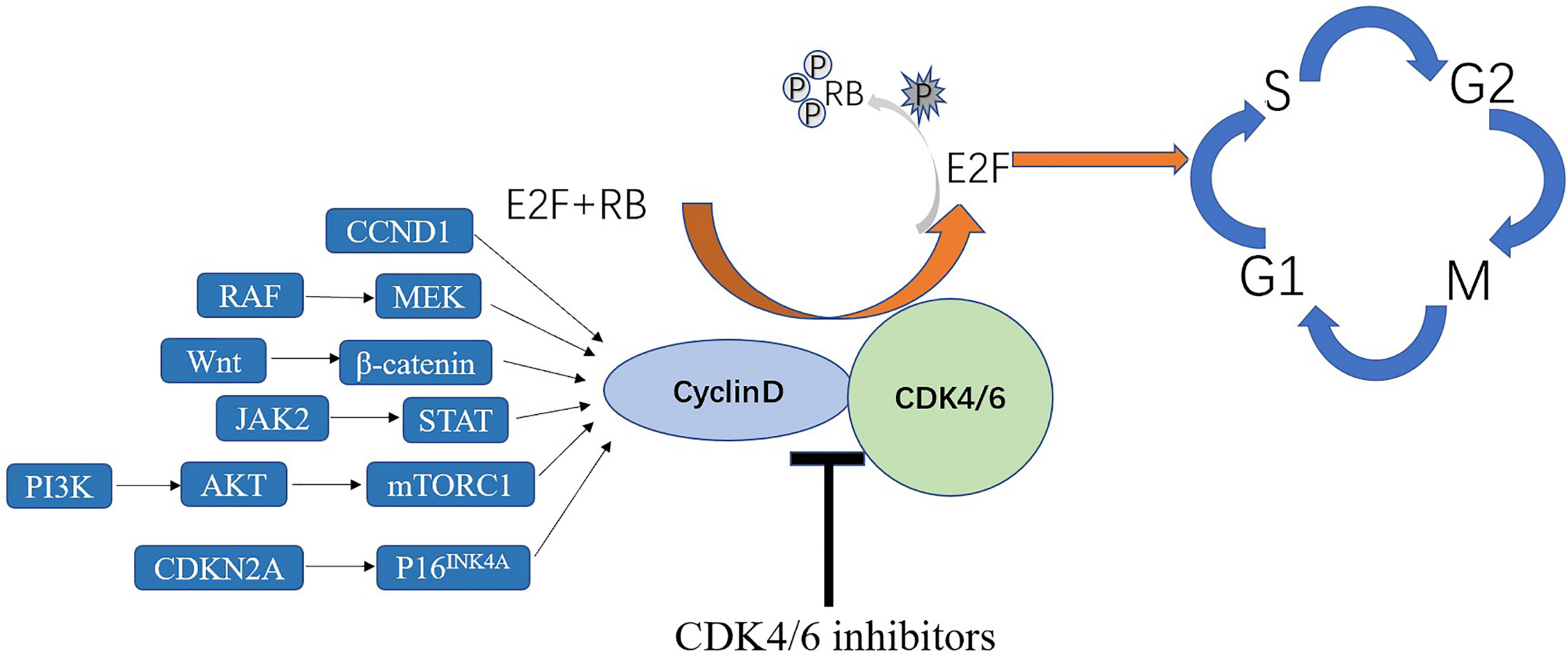
The role of CDK4/6 inhibitors in the cell cycle.

CDK4/6 inhibition has been tested in several clinical trials as a plausible treatment option for lung cancer (11–14). CDK4/6 inhibitors, designed to inhibit uncontrolled cellular proliferation, made tumor types with better efficacy and few adverse effects in which CDK4/6 plays a key role in G1-to-S-phase cell-cycle transition to be targeted (9). It can inhibit tumor growth by decreasing phosphorylation of retinoblastoma (RB) protein and inducing cell cycle arrest at the G1/S phase transition, inducing irreversible growth arrest or cell death when used alone or in combination with other therapies (15, 16) (**Figure 2**) and also promote anti-tumor immunity (17). Some drugs have been approved by the Food and Drug Administration (FDA) as treatment agents to be combined with letrozole in the treatment of hormone receptor (HR)-positive advanced-stage breast cancer (18, 19). Some clinical trials of CDK4/6 inhibitors in other tumors have achieved initial impressive results (20). CDK4/6 inhibitors are still in the early stage of other cancers, mainly confined to basic experiments and stage I or II clinical trials, such as liposarcoma, lymphoma and many other advanced cancers (21–23). A study indicated that CDK4/6 inhibitors in patients with head and neck squamous cell carcinoma have the objective response rate of 39% (n = 62) (24). Some cell cycle inhibitors also have been used in human clinical trials and achieved success in lung cancer (25, 26). Therefore, this article reviews the mechanisms of CDK4/6 inhibitors in NSCLC, monotherapy using CDK4/6 inhibitors, and the effects of combining them with other drugs in the context of NSCLC treatment.

## Monotherapy of CDK4/6 Inhibitors in NSCLC

With the recent development of highly specific CDK4/6 inhibitors (Palbociclib, Ribociclib, and Abemaciclib) and the approval of their use by the FDA for advanced metastatic breast cancer, designing multiple clinical trials using these agents for lung cancer have attracted great interest (9). However, effective strategies for formulating appropriate trial designs have not been determined. Thus, proper experiments in suitable animal models and clinical trials are needed. Palbociclib was approved in 2016 in terms of structure, and ribociclib and palbociclib are extremely similar. An *in vitro* study showed that the inhibitory effects of ribociclib and abemaciclib on CDK4 are stronger than the inhibitory effect of CDK6 and palbociclib is similar (27). Current clinical studies on NSCLC mainly focus on the phases I and II clinical studies of palbociclib and abemaciclib (**Table 1**).

**Table 1:** Clinical trials of CDK4/6 inhibitors in NSCLC (Has results).

CDK4/6 Inhibitor	Trial ID	Design	Status	Phase	Condition or Disease	Genetic Alternation Criteria	Median PFS*/m	Median OS*/m
Palbociclib	NCT01291017	P*	Completed	II	stage IV NSCLC	CDKN2a (p16)	3.2	7.7
						protein absent		
	NCT02154490	P* *vs* Docetaxel	Active, not recruiting	II	recurrent or stage IV squamous NSCLC	positive for CDK4,	1.7 *vs* UK	7.1 *vs* UK
						CCND1,CCND2,		
						and CCND3		
Abemaciclib	NCT02152631	A* *vs* Erlotinib	Active, not recruiting	III	stage IV NSCLC (KRAS mutation)	Kras 12 or 13 mutations	3.6 *vs* 1.9	7.4 *vs* 7.8
	NCT02411591	A* + Necitumumab	Completed	Ib	advanced NSCLC	\\	2.14	6.93
	NCT02450539	A* + Docetaxel	Completed	II	NSCLC stage IV	\\	2.5	7
		A* + Pemetrexed					#5.6 (A +P#)	
		A*+ Gemcitabine						
	NCT02079636	A* + Ramucirumab	Completed	I	NSCLC	\\	1.6 (A +G) 4.8 (A + R)	\\
		A* + LY3023414						
		A* +Pembrolizumab						

PFS*, progression free survival; OS*, overall survival; P*, Palbociclib, A*, Abemaciclib; P#, pemetrexed; G, gemcitabine; R, ramucirumab; \\ , not mentioned.

That NSCLC tumor actively targets the CDKN2a/p16 locus rather than the observed mutational enrichment in this locus due to a selection process during lung carcinogenesis and tumor progression. Hence, several clinical studies have been conducted.

### Palbociclib

Palbociclib is a unique selective and promising inhibitor of CDK4 and CDK6 and a cell permeable pyridopyrimidine with oral bioavailability (20, 28). Although CDK4/6 can bind with cyclin D1, resulting in Rb hyperphosphorylation, palbociclib can block Rb phosphorylation and prevent E2F1 release by separating CDK4/6–cyclin D1 complexes, resulting in G1 phase arrest and inhibit tumor growth (29). A phase II clinical study of palbociclib included 19 patients with advanced NSCLC previously treated with p16-null staining and immunohistochemistry, and tumor progression was documented (30). There were 16 evaluable patients who had no objective response, and eight (50%) patients were stable for 4.0–10.5 months. The median progression-free survival (PFS)was 3.2 months, and median overall survival (OS) was 7.7 months. The results showed that palbociclib alone was mainly used as a cell inhibitor inducing aging, but not apoptosis, and the median PFS was equivalent to other available second-line chemotherapeutic drugs (31) and PD-1 inhibitors (32, 33). In addition, the reduction rate of grade 3/4 cytopenia with palbociclib in the treatment of NSCLC was 16%, which was better than that of many effective chemotherapeutic drugs for second- or third-line therapy. A Lung-MAP trial (SWOG S1400) demonstrated the amplification of CDK4 or CCND1/2/3 in patients with squamous NSCLC and tumor. Of the 32 patients included in this study, only two (6%) had a partial response and 38% were stable. The median PFS was 1.7 months, and the median OS was 7.1 months. Unfortunately, in these genomically selected patients, palbociclib did not demonstrate any antitumor activity (12). A phase II pragmatic basket trial demonstrated antitumor activity of palbociclib in patients with NSCLC with CDKN2A alterations. Of the 29 patients who were enrolled, one patient had partial response and six patients with SD were observed, for a disease control rate of 31%. The median PFS was 8.1 weeks, and the median OS was 21.6 weeks. There were 11 patients who had at least one grade 3 or 4 adverse event (AE) or serious AE (SAE) possibly related to palbociclib (most common, cytopenias) (34).

### Abemeciclib

As an effective and selective small-molecule inhibitor of CDK4 and CDK6, abemaciclib has a wide range of antitumor activity in preclinical models and acceptable toxicity profile in animals such as mice. Preclinical data showed that the sensitivity of KRAS-mutant NSCLC xenograft models to abemaciclib was higher than that of wild-type KRAS gene expression model (13). Moreover, a JPBA phase I study showed that a single-agent abemaciclib has acceptable tolerability or safety and presented evidence of clinical activity in patients with heavily pretreated metastatic NSCLC (35). In addition, they demonstrated that the combined use of ramucirumab and abemaciclib is consistent with the safety profile of single-agent abemaciclib, with lower hematologic toxicity. The total incidence of neutropenia was 23%, and the incidence of grades 3–4 neutropenia was 10%. A phase III JUNIPER clinical trial was designed according to the result of these studies. In this trial, 453 patients who had stage IV NSCLC with KRAS mutations (codon 12 or 13) and disease progression after two lines of therapy were randomized in a ratio of 3:2 into abemaciclib and erlotinib groups (including a platinum-based regimen). The median OS was similar in both groups (7.4 *vs*. 7.8 months; HR, 0.97; 95% CI, 0.77–1.22; p = 0.77), and the median PFS was significantly better in the abemaciclib group (3.6 *vs*. 1.9 months; HR, 0.58; 95% CI, 0.47–0.72; p <0.001). The response rate (8.9% *vs*. 2.7%; p = 0.01) and disease control rate (54% *vs*. 32%; p <0.001) were significantly better in the abemaciclib group. In this study, compared with erlotinib, the OS in stage IV NSCLC patients harboring KRAS mutations did not improve. However, the additional studies of abemaciclib in other NSCLC subpopulations or in combination with other drugs are required to increases in response rates and PFS (13).

## Combination of CDK4/6 Inhibitors and Other Anti-Lung Cancer Therapies

The disappointing results of palbociclib and abemaciclib in NSCLC clinical trials have prompted studies on the effect of combination of CDK4/6 inhibitions and other therapies. Owing to the unsatisfactory results of single-drug treatments, in-depth study of the pathogenesis of NSCLC, and increasing treatment methods for NSCLC, combinations of CDK4/6 inhibitors have been extensively studied.

### Combination of CDK4/6 Inhibitors and Chemotherapy

CDK4/6 inhibitors and chemotherapeutic drugs may have antagonistic effects. For example, CDK4/6 inhibitors in combination with gemcitabine improved antitumor activity without G1 cell cycle arrest in calu-6 xenografts tumor-bearing mice (36). However, another study demonstrated combinations of palbociclib and taxanes at clinically available doses in multiple SqCLC models enhanced antitumor effects by destroying the pRB-E2F signaling pathway (37). Based on preclinical data, a phase Ib clinical study tested abemaciclib in combination with pemetrexed, gemcitabine, or ramucirumab in patients with metastatic NSCLC and confirmed the safety and tolerability of these combinations in previously treated unselected patients with advanced/metastatic NSCLC (38). In these patients, the all-cause high grade (3/4) fatigue occurred in 17–25%.High-grade diarrhea can be well controlled by antidiarrheal treatments and/or dose adjustments.

### Combination of CDK4/6 Inhibitors and Immune Checkpoint Inhibitors

The emerge as the times require of immune checkpoint blockade immediately led to the studies of the possible interactions of these therapies with CDK4/6 inhibitors. CDK4/6-targeted therapies have a complex network of immunomodulatory effects on tumor cells and their tumor microenvironment (39). The addition of CDK4/6 inhibitor to chemotherapy/ICI regimens in murine syngeneic tumor models enhanced antitumor response and overall survival compared with chemotherapy, and ICI combinations alone and transient exposure of CDK4/6 inhibition in patients with SCLC during chemotherapy treatment enhanced immune system function by preserving peripheral lymphocyte counts and enhancing T-cell activation (17). These results showed the synergistic antitumor effect of CDK4/6 and immune checkpoint-related inhibitors. The mechanism of CDK4/6 inhibitors combined with immunotherapy may be as follows: First, CDK4/6 inhibitors decrease promoter hypomethylation and inhibit E2F release by inhibiting the proliferation of regulatory T (Treg) cells and the expression of DNA methyltransferase in Treg cells (17). Furthermore, CDK4/6 inhibitors promote tumor cell clearance by enhancing cytotoxic T cells (CTLs) to kill tumor cells (40). Finally, the cyclin D1–CDK4 complex directly phosphorylates speckle-type POZ protein (SPOP), and CDK4/6 inhibitors can enhance the immune escape of tumors by reducing the ubiquitination of SPOP and the degradation of PD-L1 (41). Preclinical research showed that CDK4/6 inhibitors in combination with anti-PDL1 antibodies promote tumor regressions and the effect is accompanied by enhanced antigen presentation, T cell inflamed phenotype, and cytotoxic T cell-mediated clearance of lung cancer cells; moreover, this combination improves the overall survival rates in mouse tumor models (17, 40, 42). All of these mechanisms provide a theoretical basis for the combination therapy of CDK4/6 inhibitors and immune checkpoint inhibitors in NSCLC in the future and related clinical trials (NCT03601598) are ongoing at present (43).

### CDK4/6 Inhibitors as Radiosensitizers

Multiple preclinical and small sample clinical studies showed that CDK4/6 inhibitors exhibit a collaborative effect during radiotherapy *in vitro* and *in vivo* and show well-tolerated toxicity and promising efficacy in patients (44–47). The potential mechanisms of clinical radiosensitization effects might be apoptosis enhancement, cell cycle progression blockage, and induction of cellular senescence and antitumor immunity (48). A preclinical study showed that abemaciclib and ionizing radiation (IR) had a good radiosensitization effect on tumor cells in proliferative and plateau-phase and tumor xenografts, but had little radiosensitization effect on normal cells, and improve the radiation sensitivity of NSCLC *in vitro* and *in vivo*. Abemaciclib inhibited IR-induced DNA damage repair and caused RB-dependent cell cycle arrest; furthermore, the study identified possible predictive biomarkers (p53, RB, and SDF-1) to guide the efficacy and efficacy of the combination therapy, emphasized that CDK4/6 axis is a potential radiation target for NSCLC and warranting the value of abemaciclib as a radiation modifier in clinical trials (49). Therefore, CDK4/6 inhibitors may have different radiosensitization effects in NSCLC, and its mechanisms need to be further assessed. However, most clinical trials of combination therapies are still in the recruitment stage and further work is needed to find the best combination of radiotherapy drugs.

### Combination of CDK4/6 Inhibitors and Other Anti-Lung Cancer Drugs

Combinations of CDK4/6 inhibitors and other targeted drugs have broad prospects. PI3K-AKT-mTOR and RASRAF-MEK-ERK pathway inhibitors showed synergistic tumor inhibition in many preclinical *vitro* and *vivo* models in NSCLC with CDK4/6 inhibitors (50–52). Palbociclib sensitizes lung cancer cells to EGFR-TKI and gefitinib (25). In addition, the combination of MEK inhibitor (trametinib) and palbociclib has significant anti-CDKN2A-mutant and anti-KRAS-mutant NSCLC activities in preclinical models (53). Moreover, in view of the key role of mTOR in cell growth and proliferation, mTOR inhibitors are considered as good candidates for synergism action with CDK4/6 inhibitors. Combinations of CDK4/6 inhibitors and mTOR inhibitors can enhance growth inhibition and induction of apoptotic cell death in p16-null NSCLC cells (30). A recent study also demonstrated that combined treatment with the CDK4/6 inhibitor and a novel distinctive structure PI3Kα inhibitor through arrest enhancing G1-phase and enhancing inhibition of Rb phosphorylation to against KRAS-mutated NSCLC (54). In addition, several on-going clinical trials are studying advanced NSCLC associated with the combination of CDK4/6 inhibitors with ERK, MEK, or mTOR inhibitors (NCT03170206, NCT02065063, NCT02857270, and NCT03454035) based on these and other promising preclinical data (3).

## Prospects and Future Application

CDK4/6 inhibitors may exert an essential role in the treatment of NSCLC. Although some phase I/II clinical trials of CDK4/6 inhibitors in patients with advanced/metastatic lung cancer have not yet achieved positive results, which may be related to the small sample size of clinical trials and the lack of effective biomarkers. Given the preclinical benefits of CDK4/6 inhibitors in molecularly selected subsets, CDK4/6 inhibitors may have another role in the treatment of NSCLC in selected populations based on reasonable biomarkers, combined with radiotherapy and other agents, including growth factor pathway inhibitors and immune checkpoint inhibitors. Besides, the mechanism of CDK4/6 inhibitor resistance and the identification of sensitive predictive markers have also been reported, including acquired RB1 mutations, loss of RB1, loss of function mutations of FAT-1, CCNE1 overexpression, CDK6 overexpression, CCNE1/RB1 ratio, interferon β expression (55), CDK4 phosphorylation and tumor cloning kinetics (2, 56, 57). Therefore, the clinical efficacy of CDK4/6 inhibitors in NSCLC depend on the development of predictive biomarkers and biologically rational combination therapy, which might include the addition of growth factor pathway inhibitors in patients with signal transduction pathway mutations or the addition of immune checkpoint inhibitors in patients with immunostimulatory tumor phenotypes. Based on these, more basic and clinical studies are needed explore the precise beneficiaries of CDK4/6 inhibitors in NSCLC treatment in the future.

## Author Contributions

JZ, DX, and YZ collected the references and wrote the manuscript. All authors contributed to the article and approved the submitted version. ZZ and XY acquired funding and supervised this study.

## Funding

This work was supported by National Natural Science Foundation of China (No. 81872461).

## Conflict of Interest

The authors declare that the research was conducted in the absence of any commercial or financial relationships that could be construed as a potential conflict of interest.

## Publisher’s Note

All claims expressed in this article are solely those of the authors and do not necessarily represent those of their affiliated organizations, or those of the publisher, the editors and the reviewers. Any product that may be evaluated in this article, or claim that may be made by its manufacturer, is not guaranteed or endorsed by the publisher.
